# Charcot-Marie-Tooth Disease With Severe Hand Contractures and Respiratory Failure Requiring Long-Term Ventilator Support: A Case Report

**DOI:** 10.7759/cureus.92525

**Published:** 2025-09-17

**Authors:** Pavani Battula, Hammad Raza

**Affiliations:** 1 Internal Medicine, Fox Subacute Care Center, Mechanicsburg, USA

**Keywords:** brainstem stroke, charcot-marie-tooth disease, inherited peripheral neuropathy, palliative care, respiratory failure

## Abstract

Charcot-Marie-Tooth (CMT) disease is the most common hereditary motor and sensory neuropathy, predominantly affecting distal limb muscles, particularly in the lower extremities. Respiratory muscle involvement is rare and not typically associated with ventilator dependence. We report a case of a 76-year-old man with a longstanding history of CMT who developed severe bilateral hand contractures and progressive respiratory failure following a brainstem stroke. Despite multiple weaning attempts, he remained ventilator dependent due to neuromuscular weakness and recurrent respiratory infections. This case highlights the importance of vigilant respiratory monitoring in advanced CMT, especially in the context of comorbid neurologic events.

## Introduction

Charcot-Marie-Tooth (CMT) disease is among the most prevalent inherited neuropathies, affecting approximately one in 2,500 individuals globally [1]. It is characterized by slowly progressive distal muscle weakness, sensory loss, and foot deformities, such as pes cavus and foot drop [2]. It is also associated with reduced or lost deep tendon reflexes. While CMT is often associated with a slow, progressive course and preserved life expectancy, complications involving the upper extremities and respiratory muscles are less commonly reported [1,2]. Here, we present a rare case of an elderly patient with advanced CMT who developed severe hand contractures and ventilator dependence after a brainstem stroke.

## Case presentation

Our patient, a 76-year-old man, was diagnosed with CMT type 1 at the age of nine years when he had difficulty running and an increased frequency of falls. His family history included a father and one sibling with similar symptoms, suggesting an autosomal dominant pattern.

**Figure 1:**
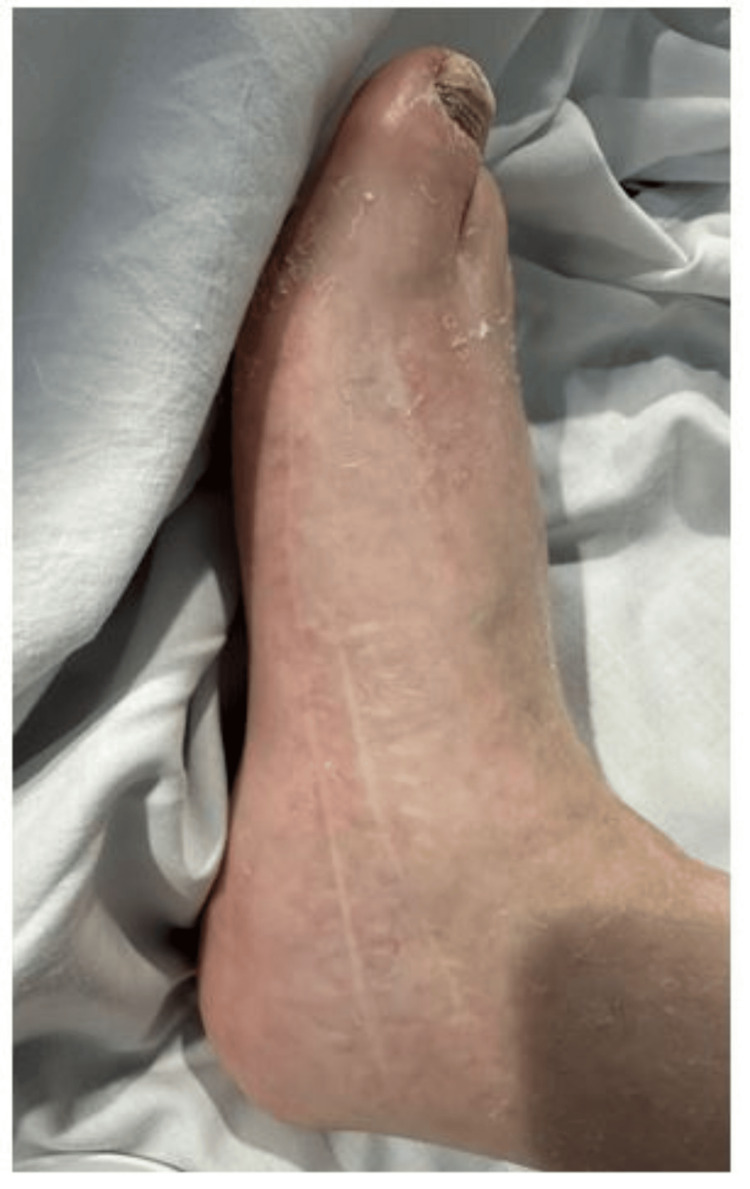
Postoperative image following foot surgery for Charcot-Marie-Tooth disease-related deformity.

**Figure 2:**
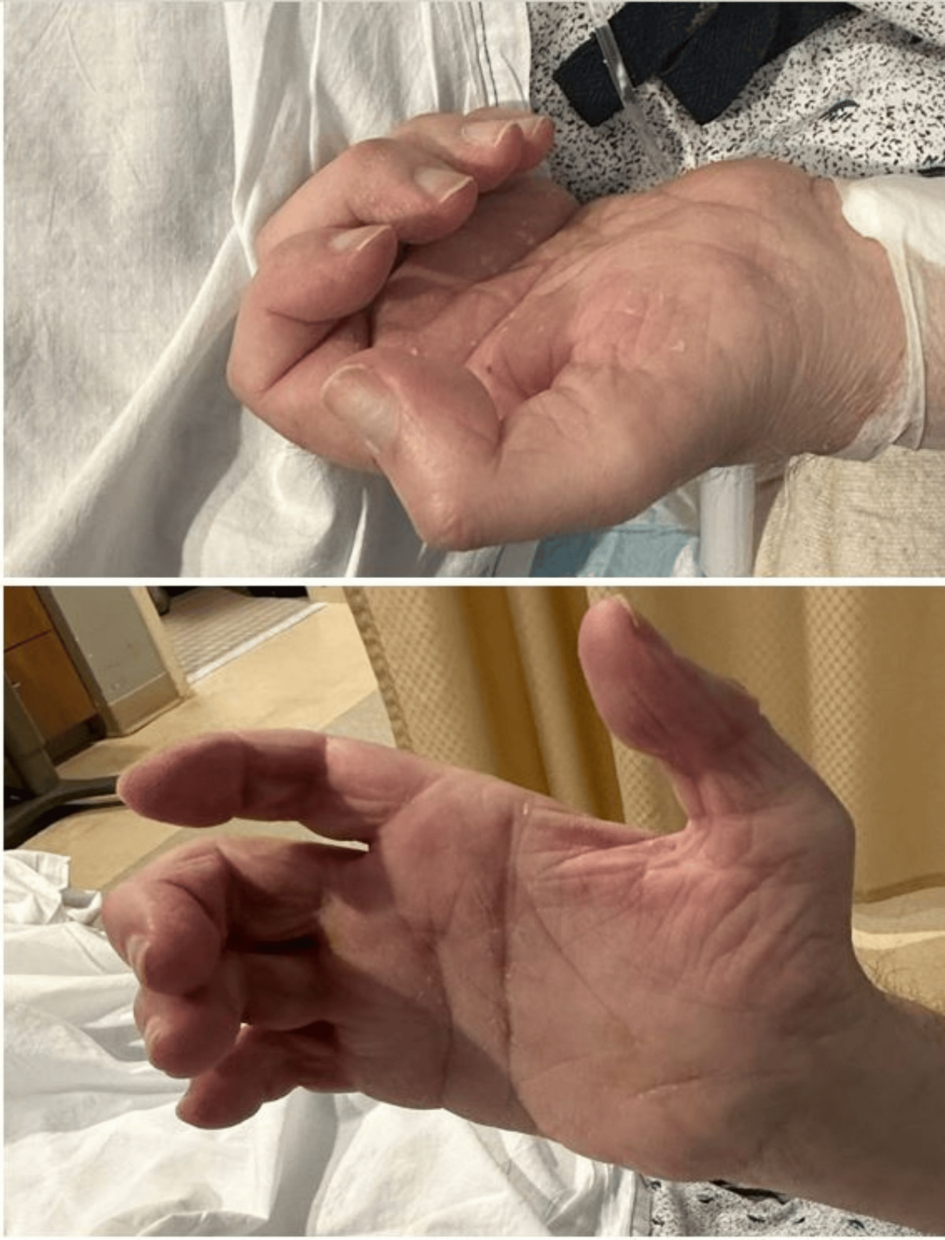
Bilateral hand contractures showing fixed flexion deformities typical of advanced peripheral neuropathy.

He experienced progressive foot weakness over the decades, requiring bilateral corrective foot surgeries in his 50s, as shown in Figure 1. Over the last decade, he developed progressive hand weakness, culminating in severe contractures of the fingers and thumbs by age 60 (Figure 2). By age 71, worsening leg weakness and poor balance rendered him non-ambulatory.

**Figure 3:**
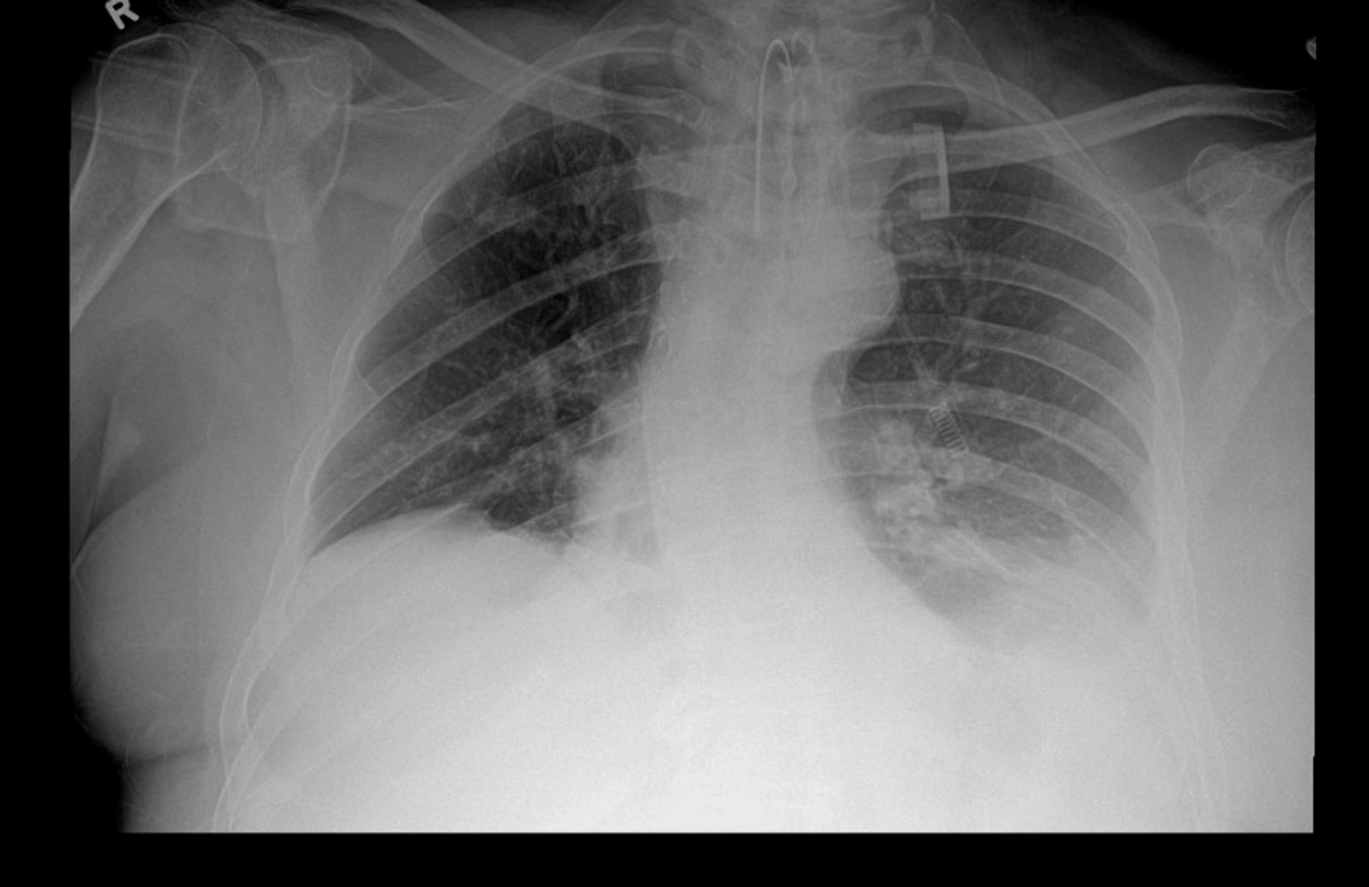
Chest radiograph demonstrating bilateral lower lobe atelectasis with elevated right hemidiaphragm and mild scoliosis, consistent with respiratory compromise in a patient with Charcot-Marie-Tooth disease.

He was no longer able to perform self-care tasks. There was no history of diabetes, autoimmune disease, or other neuromuscular conditions. In 2024, the patient suffered a brainstem stroke, leading to dysphagia and worsening dyspnea. By early 2025, he reported orthopnea and increased daytime somnolence. A chest X-ray revealed right hemidiaphragm elevation and mild thoracic scoliosis (Figure 3). Pulmonary function tests showed a restrictive pattern, and arterial blood gas analysis confirmed chronic hypercapnia.

Table 1 outlines the timeline of his clinical progression.

**Table 1:** Timeline of clinical progression.

Age (years)	Clinical milestone
9	Diagnosed with Charcot-Marie-Tooth disease type 1
50s	Bilateral foot corrective surgeries
60	Developed bilateral hand contractures
71	Became non-ambulatory
75	Brainstem stroke, onset of respiratory symptoms, and intubation
76	Ventilator dependence

Initially managed with non-invasive ventilation, he rapidly progressed to requiring intubation at age 75. Multiple extubating attempts were unsuccessful due to poor cough effort, mucus retention, and repeated episodes of pneumonia. Ultimately, a tracheostomy was performed, and he remained on full-time mechanical ventilation.

Despite these complications, the patient remained mentally intact, engaged, and communicated effectively with staff. He had a history of tobacco use in early adulthood and drank socially. Before the loss of mobility, he led an active life managing a campground and enjoying hunting.

## Discussion

CMT is a hereditary peripheral neuropathy, first described by Jean-Martin Charcot and Pierre Marie of France in 1863 and Howard Henry Tooth of the United Kingdom in 1886 [3].

There are three main modes of inheritance in CMT gene mutations: dominant, recessive, and X-linked [4], as shown in Table 2. Autosomal dominant indicates that one gene from either parent causes the disease, with a 50% chance of passing it on to a child. With autosomal recessive inheritance, if both parents are carriers, then their child has a 25% probability of developing the disease. X-linked CMT involves the X chromosome, which in turn affects a child's biological sex. A boy has a 50% chance of inheriting X-linked CMT from his mother. Symptoms differ depending on the CMT type, age at onset, and whether the axon or myelin is involved [5].

**Table 2:** Summary of types of Charcot-Marie-Tooth (CMT) disease. Table created by the authors.

CMT type	Genetic cause	Pathology	Inheritance	Age of onset	Common symptoms	Severity/notes
CMT1A	PMP22 gene duplication	Demyelinating	Autosomal dominant	Early childhood	Foot drop, lower limb weakness	Most common form
HNPP	PMP22 gene deletion	Demyelinating	Autosomal dominant	Variable	Numbness, tingling from minor nerve compression	Mild, pressure-sensitive neuropathy
CMT1B	MPZ gene mutation	Demyelinating	Autosomal dominant	Variable	Similar to CMT1A	Variable severity
CMT2	Axonal damage (various genes)	Axonal	Autosomal dominant	Variable	May include speech and breathing difficulties	Less common
CMT4	Various gene mutations	Demyelinating or axonal	Autosomal recessive	Early childhood	Severe weakness, wheelchair by adolescence	Severe, early onset
CMTX	Mutation on the X chromosome	Demyelinating	X-linked	Childhood to teen	More severe in boys; girls may be asymptomatic	Boys are symptomatic; girls are often carriers

The most common form of the disease, CMT1A, affects myelin due to an extra copy of the PMP22 gene, leading to symptoms such as foot drop and weakness, often beginning in early childhood. Hereditary neuropathy with pressure palsies (HNPP), also related to PMP22, follows a one-copy deletion and results in numbing and tingling from mild nerve compression.

CMT1B, also a demyelinating form, is associated with mutations of the MPZ gene and has a variable age of onset, like CMT1A. CMT2 is characterized by direct axonal involvement and is less frequent, with symptoms that include speech and breathing difficulties.

CMT4 is an autosomal recessive and severe early-onset form, often leading to wheelchair-bound status by adolescence. Finally, CMTX is transmitted in an X-linked manner, more severe in boys since, compared to the girls, they have only one X chromosome; the girls could have a milder form of the disease and be only carriers with no disease.

Sometimes, a new genetic mutation occurs during early development, and the child develops CMT with no previous family history of the disease.

CMT, however, is typically associated with distal lower extremity involvement (the feet, ankles, and toes) [2]. Distal lower limb contractures are characteristic [2], and this fact must be kept in mind when seeing patients. Respiratory failure due to diaphragmatic involvement occurs in fewer cases, often as a late manifestation [5]. But while rare, contractures in the upper limbs and respiratory complications are increasingly recognized as stages of advanced disease [6]. Weakness in the upper extremity, especially in the intrinsic muscles of the hand, is reported to occur in patients with CMT1 by adulthood [7]. The resulting respiratory failure in our patient was due to many factors. Distinguishing complications due to CMT progression from those exacerbated by the brainstem stroke is challenging, but both likely influenced the patient’s clinical course. He had pre-existing diaphragm weakness from CMT, which was then made worse by the bulbar dysfunction brought about by a brain stem stroke. His case provides a nice illustration of the way that neuromuscular disease can progress, but also how there are other problems on top of this, which lead to complications.

This case highlights the importance of proactive respiratory evaluation in patients with longstanding neuropathies. Monitoring lung function early with baseline spirometry and polysomnography helps in the early detection of declining respiratory capacity and nocturnal dyspnea, respectively. This preventive strategy can help preserve the quality of life in CMT patients. Early rehabilitation post stroke can help decrease morbidity.

Furthermore, timely palliative discussions regarding goals of care are essential in managing quality of life.

## Conclusions

Although CMT is often viewed as a peripheral motor neuropathy with slow progression, it can lead to disabling complications. These are associated with ascending contractures in the hands and muscle weakness, and to an extent, involvement of the respiratory muscles, leading to inadequate ventilation. Respiratory failure, although rare, is a potentially life-threatening complication, especially in patients with distinct genetic subtypes or comorbid conditions. Additionally, neurological events such as strokes can further compromise neuromuscular function, worsening mobility and respiratory capacity. A multidisciplinary approach that includes neurology, rheumatology, pulmonology, and physical therapy can help lower the disease burden and functional dependence in affected individuals, even in the setting of advanced or atypical disease presentations.

## Appendices

Patient perspective

Throughout his illness, the patient remained candid and thoughtful about his journey. He expressed a strong desire, assuredly, that his life could be prolonged utilizing machines only if recovery was likely. He said:

"I’ve carried this disease my whole life, and I’ve watched my father bear it too. Through it all, I’ve thrived, laughed, worked, and raised a family. But now, I’m not living anymore. I’m just surviving, day by day. And if the time comes when machines are the only thing keeping me going, I don’t want that. Not if I can’t feel the joy of being alive."

His words remind us how it feels for those of us who are chronically ill and highlight the necessity to coordinate care with personal values.
